# Intravitreal dexamethasone implant for the treatment of cystoid macular oedema associated with acute retinal necrosis

**DOI:** 10.1186/s12348-016-0116-x

**Published:** 2016-12-23

**Authors:** Parthopratim Dutta Majumder, Jyotirmay Biswas, Asra Ambreen, Rowayda Amin, Zahedur Rahman Pannu, Ahmed Magdy Bedda

**Affiliations:** 1 Medical and Vision Research Foundations, Sankara Nethralaya, 18, College Road, Chennai, 600 006 Tamil Nadu India; 2 Ophthalmology department, Faculty of Medicine, Alexandria University, 42 Memphis street, Camp Caesar, Apt #5, Alexandria, Egypt; 3 Bangladesh Eye Hospital, Dhaka, Bangladesh

**Keywords:** Acute retinal necrosis, Cystoid macular oedema

## Abstract

**Background:**

Acute retinal necrosis is a rare but devastating ocular condition. We report two cases of acute retinal necrosis in immunocompetent patients, complicated by cystoid macular oedema and treated with intravitreal dexamethasone (OZURDEX®) implant.

**Results:**

Two patients diagnosed with acute retinal necrosis were treated with intravenous acyclovir. Both of them developed cystoid macular oedema following resolution of viral retinitis. Ocular condition of the first patient was further complicated by central serous chorioretinopathy. Under unavoidable circumstances, cystoid macular oedema in both the patients was treated with intravitreal dexamethasone implant with great caution. Resolution of cystoid macular oedema without recurrence of viral retinitis was noted in the long-term follow-up.

**Conclusions:**

Findings of the case report should be interpreted cautiously, and extreme caution should be exercised prior deciding the management with a corticosteroid implant in patients with viral retinitis. However, intravitreal dexamethasone implant can be a useful option in selected patients with cystoid macular oedema in acute retinal necrosis.

## Findings

### Introduction

Acute retinal necrosis (ARN) is an uncommon, devastating, and potentially blinding necrotizing retinitis. It is most commonly seen in healthy young individuals, but can be seen in immunocompromised patients also [1]. ARN is a clinical diagnosis, and polymerase chain reaction of the vitreous or aqueous aspirate can be used to identify the viral DNA responsible for the disease [1]. The major causes of poor visual outcome in a case of ARN are retinal detachment, optic nerve, or macular involvement by ischemic vasculopathy [2]. Involvement of the posterior pole is relatively uncommon, except for inflammatory macular oedema, epimacular membrane formation, or retinitis in untreated or late stages of the disease. Cystoid macular oedema in these patients tends to be recurrent and resistant to treatment, threatening central vision even after resolution of ocular inflammation and requires additional treatment [3].

We report two cases of ARN in immunocompetent patients, complicated by cystoid macular oedema and treated with intravitreal dexamethasone (OZURDEX®) implant.

### Case report

#### Patient 1

A 36-year-old, otherwise healthy woman presented to us with sudden painless reduction of vision in her left eye since 1 month. She was diagnosed with ARN elsewhere 20 days back and was on oral valacyclovir 1 g twice a day and oral steroid 20 mg/day.

**Fig. 1:**
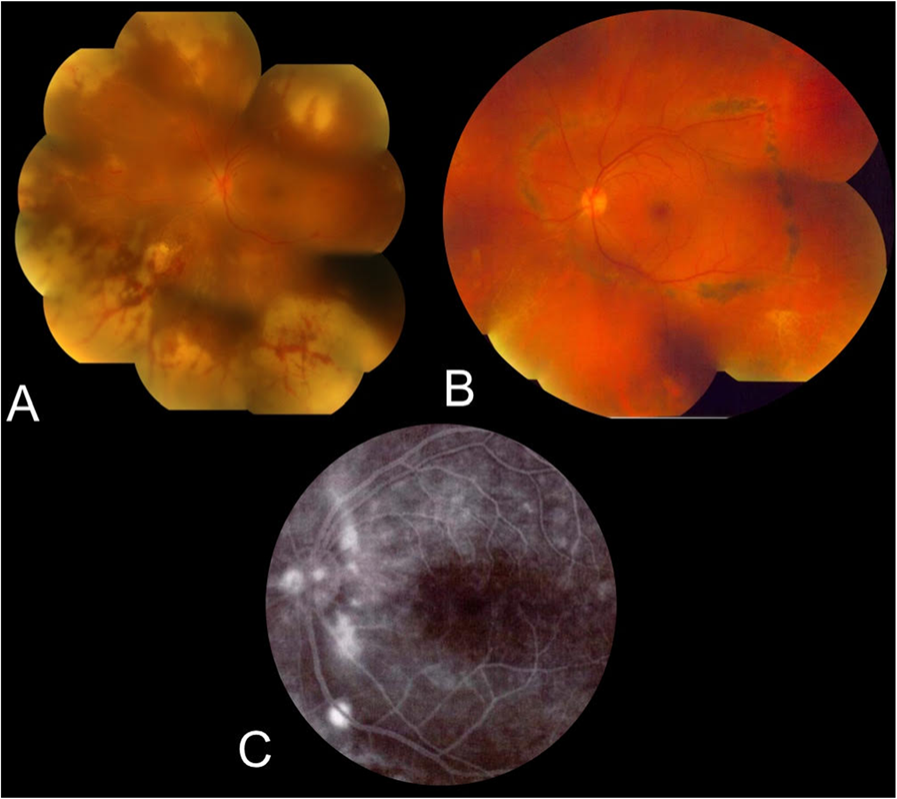
Left eye of a 36-year-old lady with acute retinal necrosis. **a**. Color fundus pictures at the initial presentation. **b**. Healed retinitis with prophylactic laser treatment. **c**. Fundus Fluorescein Angiography showing leak in the inferotemporal quadrant

On examination, her best corrected visual acuity (BCVA) was 6/6, N6 in the right eye and 6/18, N 36 in the left eye. Slit lamp and fundus examination was essentially normal in the right eye. The left eye showed 1+ anterior chamber cells and flare. Fundus examination of the left eye showed moderate vitritis and resolving necrotizing retinitis (Fig. 1a). Aqueous aspirate, obtained via anterior chamber paracentesis, was sent for PCR analysis and found to be positive for *varicella zoster* viral genome.

The patient was started on intravenous acyclovir 500 mg three times a day. Subsequently, she was started on oral corticosteroid 40 mg/day. After 3 weeks of intravenous therapy, oral valacyclovir 1 g three times was started with tapering of oral steroids. Within 3 weeks, with resolving vitritis and retinitis, her BCVA improved to 6/7.5, N10. Prophylactic laser treatment was applied on the normal retina to surround the posterior edge of the necrotic retina by rows of confluent and circumferential laser burns (Fig. 1b).

Her condition remained stable for 3 months when she came back with a drop in vision, and her BCVA in left eye was 6/9, N10. Fundus examination revealed cystoid macular oedema (CME) in the left eye, which was confirmed in optical coherence tomography (OCT) with thickness of 422 μ. She was started on topical nepafenac, and dose of oral steroid was hiked up to 50 mg/day.

She came back after a month with sudden drop in vision in her left eye. Her BCVA in the left eye was 6/36, N12. Fundus examination of the left eye revealed pocket of subretinal fluid in the inferotemporal quadrant. Fluorescein angiography (FFA) confirmed the presence of central serous chorioretinopathy (CSR) (Fig. 1c). Optical coherence tomography (OCT) confirmed the presence of CSR with deterioration of CME in the left eye with thickness of 573 μ (figure). She was advised to stop oral steroid, and focal laser to the leak was done. She came back after a month with resolved CSR and persistent CME, and her BCVA improved to 6/12, N9 in the left eye.

**Fig. 2:**
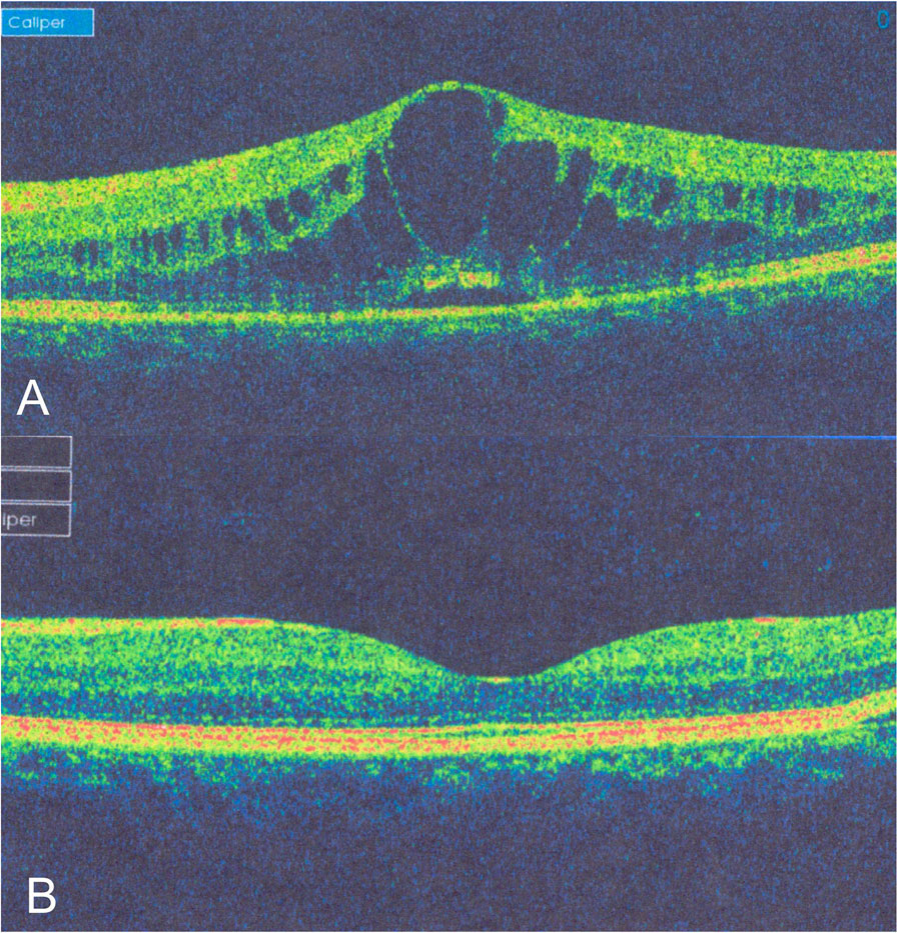
OCT of the left eye showing. **a**. Cystoid macular oedema and **b**. Resolved cystoid macular oedema after two doses of intravitreal dexamethasone implant with oral antiviral treatment

She came back with significant vitritis with persistent CME after 2 months. OCT showed deterioration of her CME with a thickness of 724 μ (Fig. 2a). Her BCVA dropped to 6/45. Intravitreal sustained-release dexamethasone implant (Ozurdex) was injected under antiviral cover in the left eye. She was kept under close follow-up and she received another injection after 3 months. Following two injections, her BCVA improved to 6/9, with marked subjective improvement in overall visual function (Fig. 2b). No reactivation of the viral retinitis was observed at the end of 2 years of follow-up. She received oral antiviral medications for 8 months following intravitreal implant administration.

#### Patient 2

**Fig. 3:**
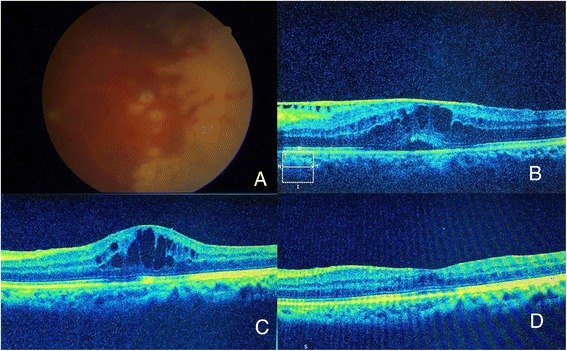
Left eye of a 39-year-old man with acute retinal necrosis. **a**: Areas of confluent necrotizing retinitis and occlusive arteritis in a patient of acute retinal necrosis. **b**: Epimacular membrane with macular edema presenting 4 months after resolution of acute phase of ARN. **c**: Cystoid macular edema causing a later drop of vision to 6/24 six months after quiescence of ocular inflammation. **d**: Resolution of macular edema demonstrated in SD-OCT 3 weeks after dexamethasone ocular implant with oral antiviral treatment

A 39-year-old, otherwise healthy man presented with reduction of vision in the left eye 2 weeks prior to presentation. On examination, his best corrected visual acuity (BCVA) was 6/24 in the left eye and 6/6 in the right. An examination of the anterior segment in the left eye showed multiple granulomatous pigmented KP and 1+ anterior chamber cell reaction with minimal flare. Fundus examination of the left eye revealed moderate vitritis, inflammatory optic disc oedema, peripheral confluent areas of retinal necrosis with hemorrhages, and vasculitis involving the retinal arterioles (Fig. 3a). An examination of the right eye was unremarkable. A diagnosis of ARN was made, and treatment with oral valacyclovir 1 g three times a day was commenced together with oral steroids 40 mg/day. Topical steroid prednisolone acetate 1% drop four times a day was started to control anterior chamber inflammation. The resolution of the retinitis was noted after 6 weeks with BCVA of 6/9. Patient was maintained on valacyclovir 500 mg BID (twice a day) and advised for follow-up.

After 4 months, he returned to the clinic reporting further decrease of vision in the previously affected eye. His BCVA in the left eye was 6/36. Fundus examination revealed some vitreous floaters and a blunted foveal reflex. No reactivation of retinitis was noted. SD-OCT revealed the formation of cystoid macular oedema with retinal thickness measuring 508 μ (Fig. 3b). The patient was commenced on oral valacyclovir 1 g tid, and advised for pars plana vitrectomy. After 3 weeks, there was a remarkable resolution of CME, and BCVA improved to 6/12. Treatment was tapered to 1 g valacyclovir, and retinitis remained quiescent over follow-up.

Two months later, patient returned with a drop in vision in the left eye. His BCVA was 6/24, N12 and SD-OCT revealed deterioration of CME with retinal thickness of 465 μ (Fig. 3c). Anti-VEGF injection was given with no improvement in BCVA or OCT 1 month later. Since he had declined all systemic forms of therapy, the patient was counselled to administer an Ozurdex injection. Valacyclovir was increased to 1 g three times day. One month after injection, his BCVA improved to 6/9 and retinal thickness reduced to 265 μ with complete resolution of CME (Fig. 3d). She was continued oral antiviral for 6 months following intravitreal injection of Ozurdex.

Three months later, she came back with a drop in BCVA and fundus examination revealed recurrence of CME in the left eye. She was treated with a repeat Ozurdex injection which resulted in resolution of CME with BCVA of 6/12 in 6 weeks.

### Discussion

ARN is a severe necrotizing vasoocclusive retinitis caused by herpes viruses. In the absence of appropriate treatment, retinitis progresses circumferentially with subsequent devastating visual outcome. Posterior pole involvement with retinitis is uncommon and includes the development of macular oedema, epimacular membranes, macular holes, and macular ischemia.

Although signs of intraocular inflammation disappear usually between 6 and 12 weeks following the onset of treatment, post ARN macular oedema can pose a chronic recurrent problem and a therapeutic challenge, with possible consequent reduction of central vision. CSR in ARN is very rare, but has been reported in literature [4].

The role of systemic corticosteroid therapy in controlling intraocular inflammation and resolution of the vitritis in ARN is generally well accepted [5]. Literature on use of intravitreal corticosteroid in ARN is sparse. In fact, local administration of corticosteroid has been implicated to reactivate viral retinitis [6–8]. However, in many of the cases reported, there were associated comorbidities and many of these patients were on immunosuppression for a long time [6]. Thus, definitive proof for potential risk of developing necrotizing retinitis following periocular or intraocular corticosteroids is yet to be ascertained.

Management of cystoid macular oedema following viral uveitis is always challenging and frustrating. There is no standard treatment regimen for uveitic macular oedema. Various available treatment modalities for uveitic macular oedema include topical, regional and systemic corticosteroids, immunosuppressive, and VEGF inhibitors. VEGF inhibitors have been tried in uveitic macular oedema with variable results [9–11]. The role of intravitreal corticosteroid in cystoid macular oedema has been well-established. However, the use of intravitreal steroid in infectious retinitis remains questionable and should not be advocated as primary treatment modality. Intravitreal triamcinolone (IVTA) in adjuvant with intravitreal foscarnet has been used to treat optic nerve oedema in a patient with acute retinal necrosis [12]. Choudhury et al. [13] treated four eyes of ARN with IVTA under oral antiviral cover in absence of new active lesions.

Our cases illustrate the clinical efficacy of Ozurdex in the treatment of CME in patients with ARN. However, extreme caution should be exercised prior deciding the management with a corticosteroid implant in patients with viral retinitis. In the absence of active necrotizing retinitis and long-term treatment with antiviral agents, Ozurdex can be a useful tool for the management of CME associated with ARN.
